# Duplex Real-Time RT-PCR Assays for the Detection and Typing of Epizootic Haemorrhagic Disease Virus

**DOI:** 10.1371/journal.pone.0132540

**Published:** 2015-07-10

**Authors:** Cyril Viarouge, Emmanuel Breard, Stephan Zientara, Damien Vitour, Corinne Sailleau

**Affiliations:** ANSES/INRA/ENVA-UPEC, UMR 1161 Virologie, 23 avenue du général de Gaulle-94700 Maisons Alfort-France; The Pirbright Institute, UNITED KINGDOM

## Abstract

Epizootic haemorrhagic disease virus (EHDV) may cause severe clinical episodes in some species of deer and sometimes in cattle. Laboratory diagnosis provides a basis for the design and timely implementation of disease control measures. There are seven distinct EHDV serotypes, VP2 coding segment 2 being the target for serotype specificity. This paper reports the development and validation of eight duplex real-time RT-PCR assays to simultaneously amplify the EHDV target (S9 for the pan-EHDV real-time RT-PCR assay and S2 for the serotyping assays) and endogenous control gene Beta-actin. Analytical and diagnostic sensitivity and specificity, inter- and intra-assay variation and efficiency were evaluated for each assay. All were shown to be highly specific and sensitive.

## Introduction

Epizootic haemorrhagic disease virus (EHDV) is a double-stranded RNA non-enveloped virus belonging to the *Reoviridae* family, genus *Orbivirus*. It is transmitted by *Culicoides* biting midges. EHDV has a 10-segmented RNA genome that encodes seven structural proteins (VP1-VP7) divided into two capsids and five nonstructural proteins (NS1-NS4, NS3a) [1–5]. The VP2 protein is exposed on the surface of the viral particle and determines the serotype. RNA segment 2, which encodes VP2, is targeted for RT-PCR serotyping.

To date, seven EHDV serotypes have been identified [6]. Some (EHDV-1 and EHDV-2) are endemic in the United States and Canada, causing severe clinical episodes with high mortality in some species of deer such as white-tailed deer [7,8]. Moreover, in 2006, an exotic strain of EHDV-6 was isolated from moribund and dead white-tailed deer (*Odocoileus virginianus*) in Indiana and Illinois. This EHDV-6 strain originates from a reassortment between co-circulating strains of serotypes 2 and 6 [9]. Several strains of EHDV-2, -6 and -7 can also lead to severe disease in cattle: in Japan (Ibaraki strain, serotype 2) [10]; in 2006 in Morocco, Algeria and Turkey (EHDV-6) [11]; in 2006 in Israel (EHDV-7) [12]; and in 2009 in Reunion Island (EHDV-6) [13]. More recently, EHDV-6 and -2 were reported in the Caribbean basin (Martinique, Guadeloupe Islands and French Guiana), while EHDV-1 was detected in French Guiana without clinical signs [14]. In western Kenya, a survey conducted between 2007 and 2010 showed the circulation of EHDV in indigenous calves [15]. Some positive samples could not be serotyped by seven serotype specific gel-based RT-PCRs [16]. The authors suggest that this is because the gel-based serotyping assays are less sensitive than the real-time group-specific RT-PCR, or because of mismatches in the VP2 primer sequences which were designed from the reference EHDV strains. Moreover, EHDV is regularly detected in areas where BTV also circulates (e.g. Reunion, French Guiana, Martinique and Kenya) [13–15,17]. The genome of these two viruses is very close, so it is important to have molecular tools able to both differentiate and serotype these viruses. In the last few years, real-time RT-PCR assays have been developed for the detection of all seven serotypes [18,19]. To our knowledge, only conventional RT-PCR assays were available up to now for virus serotyping [16,20].

This paper describes the development and validation (according to the methods described in chapter 1.1.5. of the World Organisation for Animal Health, OIE [21]) of eight duplex real-time RT-PCR assays for the detection and serotyping of each EHDV serotype (EHDV-1/-3, -2, -4, -5, -6, -7 and -8): a duplex pan-EHDV real-time RT-PCR assay that amplifies a portion of segment 9 of all EHDV strains coupled with a real-time RT-PCR that amplifies the beta-actin RNA of ruminant cells (housekeeping gene) and a set of seven duplex serotyping real-time RT-PCR assays that specifically amplify both the EHD segment 2 portion of each serotype and the beta-actin gene. The Ct (Cycle Threshold) value obtained with the real-time RT-PCR that amplifies the housekeeping gene is used to verify the integrity of the total RNA extracted and the absence of inhibitors that can interact during the RT-PCR steps for each biological sample tested. These assays were developed and validated to detect and serotype EHDV for application to biological samples from the field.

## Materials and Methods

### Strains and samples

#### Reference strains

The different EHDV reference strains were used to check the initial specificity of the assays.

All isolates were obtained from IEMVT-CIRAD and the Pirbright Institute.

#### Biological samples and field strains

For the diagnostic specificity evaluation, blood samples from 234 animals known to be uninfected were collected in EHDV-free areas of Metropolitan France.

All the infected biological samples used in this study were taken either from naturally infected domestic cattle or buffaloes, or from experimentally infected animals [13,14,17,22]:

40 blood samples infected by EHDV-1 (Reunion Island and French Guiana);
32 blood samples infected by EHDV-6 (Reunion Island and French Guiana);
24 blood samples infected by EHDV-6 (experimental infection).

All the Biological samples used in this study were taken during our own previous studies.

Samples from experimentally infected animals were taken during a previous study published by Breard et al [17]. All experimental protocols were reviewed by a state ethics commission and have been approved by the competent authority (Comité d’Ethique en Expérimentation Animale Val de Loire- CEEAVdL reference: 2011-09-1).

Naturally infected and non-infected samples were collected between 2009 and 2013 as part of routine veterinary investigations carried out by qualified veterinarians in the respective area of origin.

All field strains used in the study were obtained during our own previous studies and no specific passage in cells culture or in ECE were achieved for this work. Further ethical approval was not therefore needed.

The two EHDV-6 (Morocco) and EHDV-7 (Israel) field strains were provided by Biopharma and the Pirbright Institute respectively.

### Primers and Probes

#### EHDV Target

Primers and TaqMan minor groove binder (MGB) probes were designed by multiple sequence alignment of segment 9 (available in Genbank) using MegAlign 8.0.2 (Lasergene DNASTAR) for the detection of all seven serotypes and the sequences of segment 2 for serotyping. The primers and probes were selected and evaluated using Primer Express software (Primer Express Software v3.0.1 by Lifetechnologies).

#### Endogenous target

The concentration of primers and probe to detect an endogenous control (Beta actin) was optimised for use in the duplex real-time RT-PCR assays. The sequences of the two primers and probe are respectively: ACT_F_1005–1029 (5’-CAGCACAATGAAGATCAAGATCATC-3’), ACT_R_1135–1114 (5’-CGGACTCATCGTACTCCTGCTT-3’) and ACT_P_1081–1105 (VIC-TCGCTGTCCACCTTCCAGCAGATGT-TAMRA) [23].

### Nucleic Acid Sample Preparation

Total RNA was extracted from 100μl of blood samples or culture cell supernatants using the QIAcube robot (QIAGEN) or the Kingfisher 96 robot (Life Technologies) with the QIAamp Viral kit (QIAGEN; reference 52906) or the MagVet Universal isolation kit (Life Technologies; reference MV384) according to the manufacturer’s instructions.

### Duplex One-Step RT-PCR

The different primers and probe sets were used to amplify a part of the targeted gene using the AgPath One-Step RT-PCR Kit (Life Technologies; reference 4387424). The final composition of the mix used for the eight real-time RT-PCRs was optimised.

After RNA denaturation with 10% DMSO and heating at 95°C for 3 minutes, 5 μl of denaturated RNA was added to the mix and the amplifications were carried out using the same cycling parameters for all of the assays: 45°C for 10 min, 95°C for 10 min followed by 40 cycles of 95°C for 15 s and 60°C for 1 min. Fluorescence was measured during the 60°C annealing/extension step. Cycle threshold (Ct) values were measured at the point at which the sample fluorescence signal crossed a threshold value (the background level). Negative results (for assays that did not exceed this signal level) are reported as ‘No Ct’.

### Analytical Specificity

A panel of different EHDV strains including all seven serotypes was tested in each of the eight assays to check specificity. Serotypes of bluetongue, African horse sickness virus (AHSV) and other pathogens infecting cattle were also tested to ensure that there was no cross-reaction.

### Analytical Sensitivity

#### PCR detection limit (LOD_PCR_)

The detection limit of the PCR (LOD_PCR_) is the smallest number of copies of the target nucleic acid per unit volume that can be detected in 95% of cases. It was determined by three independent sessions that were conducted by testing eight replicates of three dilutions around the detection limit.

In order to evaluate the LOD_PCR_, a synthetic single strand RNA corresponding to the nucleotide sequence of each targeted amplification product was produced by *in vitro* transcription using a T7 promoter. An RT-PCR was performed with forward primers modified by addition of a T7 promoter sequence (ATT AAT ACG ACT CAC TAT AGG) [24] at the extreme 5' end of the upstream primer (no modification for the reverse primers).

The PCR product was then purified with a QIAquick PCR Purification Kit (QIAGEN). *In vitro* transcription was performed at 37°C for 4 h with a MEGAscript T7 kit (Lifetechnologies). After the transcription reaction, the template DNA was degraded by addition of 1μl of RNase-free DNase I for 15 min at 37°C. The transcribed RNA was purified with a MEGAclear kit (Lifetechnologies) and dosed by spectrophotometry.

#### Method detection limit

The method detection limit (LOD_method_) is used to estimate the smallest quantity of biological target that must be initially present in the sample for it to be detected. Two independent sessions of four extractions were performed on three quantities of synthetic RNA around the detection limit added to a negative lysed blood sample.

#### Efficiency

The efficiency of real-time RT-PCR assays was estimated on the basis of a standard curve plotting Ct values against the corresponding log ssRNA copy number per reaction. Efficiency was calculated by the formula: E% = (10^-1/slope^-1)x100.

### Diagnostic Specificity and Sensitivity

Diagnostic specificity is the proportion of samples from animals known to be uninfected that test negative in the assay. It was estimated using the blood samples from cattle sampled in EHDV-free areas of Metropolitan France. Between 92 and 138 samples were analysed for the different EHDV serotyping assays, and 234 for the pan-EHDV assay.

Diagnostic sensitivity is the proportion of samples from animals known to be infected that test positive in the assay. It was estimated using EHDV-positive blood samples from 96 animals collected in French Guiana and Reunion Island or during animal experimentation. Ninety-six samples were analysed for the pan-EHDV assay, 40 for the EHDV-1 serotyping assay and 32 for the EHDV-6 serotyping assay. No infected biological samples were available for the EHDV-2, -4, -5, -7 and -8 serotyping assays. Confidence intervals were calculated using binomial distribution [25].

### Repeatability

The repeatability of the eight real-time RT-PCR assays was evaluated by determining the intra- and inter-assay coefficient of variation (CV). A panel of eight positive samples with different viral loads (cycle threshold range 23–35) was tested in triplicate in two independent sessions. The CV was calculated for each sample [26].

Intra-assay CV = (average of each session’s Ct standard deviation (SD)/average of each session’s Ct average) x100.

Inter-assay CV = (SD of the Ct averages for each session/average of the averages for each session) x100.

## Results

### Primer and Probe Design

A comparison of segment 9 nucleotide sequences (available in GenBank) from EHDV strains isolated around the world, and representing the seven EHDV serotypes, showed a conserved sequence in the 5’ end region that allowed the design of two primers and one probe for the pan–EHDV real-time RT-PCR assay (Table 1).

**Table 1:** List of primers and probes for pan-EHDV and serotyping real-time RT-PCR assays. (W) and (E) indicate the pair of primers and/or probe that can detect “western” or “eastern” topotype.

Assay type	Primer/probe name	Sequence (5’-3’)
Pan-EHDV	EHDV_Seg9_F_7–25	AATTGCGCATGTCAGCTGC
	EHDV_Seg9_R_76–55	TTTAATTCCTCGGTCGAACGTT
	EHDV_Seg9_P_29–44	FAM-TTTGCTCGCACCCGGT-MGB
EHDV-1	EHDV1_Seg 2_F_8–31	TGTGTCAGGATGGAGGACATTAAC
	EHDV1_Seg 2_R_ 362–343 (W)	TATGCGCCTCGTCCATTCTC
	EHDV1_Seg 2_R’_ 363–341 (E)	CTATGCGTTTCATCCATTCTTGG
	EHDV1_Seg2_P_ 128–143 (E, W)	FAM-CCGCATCAAATGTATG-MGB
	EHDV1_Seg2_P’_128–141 (W)	FAM-CCGCACCAGATGTAT-MGB
EHDV-2	EHDV2_Seg2_F_1642–1665 (W)	CCTTTAAGATAAGACGGGTCGAGA
	EHDV2_Seg2_F’_1640–1666 (E)	GTCCTTTAAGGTAAGACGGGTAGAGAT
	EHDV2_Seg2_R_1798–1770 (W)	CTCAAGATATTACCGGTTAAGCATAGAGT
	EHDV2_Seg2_R’_1788–1170 (E)	TGCCGGTCATACAGAACGC
	EHDV2_Seg2_P_1728–1743	FAM-AACGAGATGTGGCTTC-MGB
EHDV-4	EHDV4_Seg2_F_2573–2591	TATCAAGCGACCCAGTCGC
	EHDV4_Seg2_R_2771–2743	CGTATGACATTCTGCAAGTCAGC
	EHDV4_Seg2_P_2635–2653	FAM-CACATCTACGGATACTGTG-MGB
EHDV-5	EHDV5_Seg2_F_403–424	ACGAATCGGAGGATACGGATC
	EHDV5_Seg2_R_522–501	TCGCGTATGATCACACTGGTCT
	EHDV5_Seg2_P_471–489	FAM-ACTATCGGTAGTGGTGTTC-MGB
EHDV-6	EHDV6_Seg2_F_560–582 (W)	GGATCTGGAACGTGCTATGATCT
	EHDV6_Seg2_F’_561–582 (E)	GTTCCGGGACATGCTATGATCT
	EHDV6_Seg2_R_734–714 (W)	CAGCCTGAATCTTCGTTTGCT
	EHDV6_Seg2_R’_734–715 (E)	CCGCCTGAATTTTTGTTTGC
	EHDV6_Seg2_P_686–702	FAM-ATAACGAACAGGGAGCC-MGB
EHDV-7	EHDV7_Seg2_F_2490–2508 (E)	CGAGAGGAACCGACCGAAG
	EHDV7_Seg2_F’_2490–2512 (W)	CGAGAAGAGCCGATTGAAGAAG
	EHDV7_Seg2_R_2627–2605 (E)	GCTTAAATGCGTATTCATGGGAT
	EHDV7_Seg2_R’_2629–2605 (W)	GTGCTTAAATGCGTATTCATAGGGT
	EHDV7_Seg2_P_2520–2537	FAM-ACTGTATGGCCGTATCTA-MGB
EHDV-8	EHDV8_Seg2_F_822–846	ACGATCCTATAATATCACGCTTGGA
	EHDV8_Seg2_R_897–878	TCTTCGATCCGCTCACTGC
	EHDV8_Seg2_P_853–869	FAM-AGCTGATGAATGGATGC-MGB

By aligning the segment 2 sequences of each EHDV serotype, it was possible to design two primers and one probe for EHDV-4, -5 and -8. There was greater sequence divergence among the different strains of serotypes EHDV-1, -2, -6 and -7, so more than two primers were designed for the specific real-time RT-PCR assays in order to ensure sensitivity.

### Real-time RT-PCR Mix

The composition of the mix after optimisation is presented in Table 2.

**Table 2:** Real-time RT-PCR mix composition.

Reagent	Volume (μl) per tube
Forward primer(s)(100μM)*	0.1
Reverse primer(s) (100μM) *	0.1
Probe (100μM) *	0.05
Pre-mix Beta- actin (2.5μM of each primer and 1.25μM of probe)	3
2x RT-PCR Buffer	12.5
25x RT-PCR Enzyme Mix	1
Nuclease-free Water	3.25

*In the case of two primers/probes, this is a 50:50 mix of each primer/probe

### Analytical Specificity

All EHDV strains (including all seven serotypes) from infected cell cultures tested positive using the pan-EHDV real-time RT-PCR assay (Table 3).

**Table 3:** Specificity of pan-EHDV and serotyping assays.

Virus Species-serotype	Origin	Reference strains	Real-time RT-PCR assays							
			Pan EHDV	EHDV1	EHDV2	EHDV4	EHDV5	EHDV6	EHDV7	EHDV8
**EHDV reference**										
EHDV-1	USA (New Jersey)	USA1955/01	11.95	14.74	No Ct	No Ct	No Ct	No Ct	No Ct	No Ct
EHDV-2	Canada (Alberta)	CAN1962/01	12.30	No Ct	15.09	No Ct	No Ct	No Ct	No Ct	No Ct
EHDV-2	Japan (Ibaraki)	JAP1959/01	11.85	No Ct	14.12	No Ct	No Ct	No Ct	No Ct	No Ct
EHDV-2	Australia	AUS1979/05	10.90	No Ct	15.92	No Ct	No Ct	No Ct	No Ct	No Ct
EHDV-3	Nigeria	NIG1967/01	15.24	15.26	No Ct	No Ct	No Ct	No Ct	No Ct	No Ct
EHDV-4	Nigeria	NIG1968/01	13.22	No Ct	No Ct	15.03	No Ct	No Ct	No Ct	No Ct
EHDV-5	Australia	AUS 1977/01	12.75	No Ct	No Ct	No Ct	12.75	No Ct	No Ct	No Ct
EHDV-6	Australia	AUS1981/07	13.67	No Ct	No Ct	No Ct	No Ct	15.87	No Ct	No Ct
EHDV-7	Australia	AUS1981/06	13.07	No Ct	No Ct	No Ct	No Ct	No Ct	11.46	No Ct
EHDV-8	Australia	AUS1982/06	13.03	No Ct	No Ct	No Ct	No Ct	No Ct	No Ct	15.25
**Field strains of EHDV**										
EHDV-1	French Guiana	EHDV1/2011.02 (4057)	17.68	17.73	No Ct	No Ct	No Ct	No Ct	No Ct	No Ct
EHDV-1	Reunion Island	EHDV1/2011.01 (6010)	14.32	15.41	No Ct	No Ct	No Ct	No Ct	No Ct	No Ct
EHDV-2	Guadeloupe Island	EHDV2/2011.02 (4976)	17.26	No Ct	19.6	No Ct	No Ct	No Ct	No Ct	No Ct
EHDV-6	Reunion Island	2003	14.15	No Ct	No Ct	No Ct	No Ct	14.38	No Ct	No Ct
EHDV-6	Reunion Island	EHDV6/2009.03	14.88	No Ct	No Ct	No Ct	No Ct	15.65	No Ct	No Ct
EHDV-6	Morocco	2006	15.02	No Ct	No Ct	No Ct	No Ct	14.56	No Ct	No Ct
EHDV-7	Israel	ISR2006/01	10.00	No Ct	No Ct	No Ct	No Ct	No Ct	13.67	No Ct

For the seven serotyping RT-PCR assays, only the homologous serotypes gave positive results (Table 3). No amplification was observed (no Ct) for BTV, AHSV or other pathogens that infect cattle (Table 4).

**Table 4:** Pathogens used to evaluate specificity.

Pathogen	Strains
Bluetongue virus (BTV)	Reference strains from South Africa BTV 1–27
African Horse sickness virus (AHSV)	Reference strains AHSV-1-9
*Chlamydophila abortus*	AB1, AB17, AC1, Mo907, OC1
*Chlamydophila pecorum*	iB3, iB5, iC2, R69, W73
*Mycobacterium*	*bovis*, *avium hominissuis*, *avium* subspecies *paratuberculosis*, *nonchromogenicum*
*Salmonella*	*enteritidis*
Rift Valley fever virus	Kenya
Mycoplasma	mmSC PG, bovis PG45, mycoïdes subspecies Capri Y-Goat, mycoïdes subspecies Capri PG3, agalactiae PG2, capricolum subspecies Capripneumoniae, arginini 2230
Toxoplasma	
Brucella	Suis 2 (Thomsen), Melitensis 1 (16M), Abortus 1 (544)

### Analytical Sensitivity

The PCR and method detection limits (LOD_PCR_ and LOD_Method_) and efficiency values for the eight real-time RT-PCR assays are presented in Table 5. The LOD_PCR_ and LOD_Method_ varied between 1 to 50 and 10 to 1,000 copies respectively. Efficiency rates were determined for the different assays on the basis of a series of dilutions of synthetic RNA. They were found to lie between 98.9 and 104.4%.

**Table 5:** Real-time RT-PCR validation.

Real-time RT-PCR assays	Pan-EHDV assays	EHDV-1/3	EHDV-2	EHDV-4	EHDV-5	EHDV-6	EHDV-7	EHDV-8
**Target**	S 9	S 2	S 2	S 2	S 2	S 2	S 2	S 2
**LOD_PCR_** (copies)	10	50	50	50	1	50	50	1
**LOD_METHOD_** (copies / 100μl blood)	500	100	1000	500	10	500	500	10
**Efficiency %**	99.9	100.2	103.5	93	98.9	100.5	104.4	99.7
**Diagnostic Specificity** (Sp)**%**	98	100	100	100	100	100	100	100
CI* at 95% of Sp	96–100 (n = 234)	97–100 (n = 135)	97–100 (n = 126)	97–100 (n = 138)	97–100 (n = 134)	96–100 (n = 92)	97–100 (n = 124)	97–100 (n = 134)
**Diagnostic Sensitivity (Se)%**	99	100	ND	ND	ND	100	ND	ND
CI* at 95% of Se	94–100% (n = 96)	91–100% (n = 40)	/	/	/	89–100% (n = 32)	/	/
**Repeatability** Intra-assay variation(CV) %	0.53–2.06	0.37–2.28	0.9–2.35	0.58–1.73	0.51–1.08	0.41–2.95	0.37–2.68	0.30–1.20
**Repeatability** Inter-assay variation (CV) %	0.51–1.83	0.13–0.96	0.58–2.69	0.06–1.20	0.98–4.16	0.05–3.84	0.05–3.47	0.50–3.67

* CI: confidence interval CV: coefficient of variation ND: Not done.

### Diagnostic Specificity and Sensitivity

Diagnostic specificity and sensitivity are 98 and 99% respectively for the pan-EHDV assay. In the absence of positive EHDV-2, -4, -5, -7 and -8 biological samples, diagnostic sensitivity is shown only for the serotyping EHDV-6 and -1 assays (100% for both assays). Diagnostic specificity was 100% for the seven serotyping assays (Table 5).

### Repeatability

The intra- and inter-assay coefficients of variation are presented in Table 5. The lowest and highest percentages are shown for each assay. All inter- and intra- assay CVs are less than 5%.

## Discussion

In order to obtain good performance for the pan-EHDV assay, we decided to target VP6 encoding gene S9. *In silico* studies of S9 sequences available in GenBank showed that nucleotide variations ranged between 1.2 and 33.9%. However, a conserved region (in the 5’ end region) appeared to be of interest for designing the primers and probe. Only one set of two primers and one probe was designed for this assay, whereas the previously reported pan-EHDV real-time RT-PCR assays use several primer pairs and/or probes targeting the NS3 or NS1 encoding genes (S10 and S5) [18,19]. The cost of the reaction can therefore be significantly reduced using this new assay. All the reference or field strains were detected and no amplification was generated with nucleic acid from the similar Bluetongue virus African Horse Sickness virus or various pathogens infecting cattle. Good diagnosis sensitivity (99%) and specificity (98%) rates were observed when testing samples from the field. Analytical sensitivity (LOD_PCR_) is very close to that reported by Schroeder *et al* (<100 copies) [26]. These authors targeted segment 5 (encoding NS1) when developing a real-time RT-PCR assay to detect all seven serotypes.

Seven real-time serotyping RT-PCR assays have also been developed and validated that target VP2 encoding gene S2, which is the primary determinant of virus serotype [6,27]. *In silico* analyses have revealed high variability of up to 30–35% between the segment 2 nucleotide sequences of different strains belonging to the same serotype (e.g. a 32% difference between the EHDV-6 of Reunion Island (Accession number HQ848379) and of Guadeloupe (Accession number HQ848380); a 32% difference between EHDV-7 Australia (Accession number AM745048) and Israel (Accession number HM156731)). Consequently, several selected primer pairs/probes were added into the same mix to be able to amplify all the different strains of EHDV serotypes 1, 2, 6 and 7 (Table 1). All seven serotype-specific assays showed good results in terms of sensitivity and specificity.

The high variability of the S2 gene underlines the need to verify for news strains that the primers/probe binding site is suitable. It also shows the need to sequence the RNA segments targeted by these real-time RT-PCRs in order to supply and complete the data bank. It might be necessary to design a new set of primers and probe.

To date, the traditional method for identifying EHDV from clinical samples is viral isolation on culture cells or embryonated chicken eggs, followed by a vironeutralisation test (VNT) using reference sera [28]. Unfortunately, reference sera are not available for each serotype. Moreover, these procedures are very time consuming (at least two weeks). To improve EHDV diagnosis, various RT-PCR assays have been developed. In the literature, only gel-based RT-PCRs are described for serotyping EHDV [16,20]. In a previous study, we described a method to easily identify EHDV serotypes using both conventional RT-PCR and sequencing [13]. To our knowledge, this is therefore the first report of real-time serotyping RT-PCR assays.

These molecular tools have been specifically developed and validated to quickly detect EHDV directly from ruminant blood. The diagnostic sensitivity and specificity of the pan-EHDV and the EHDV-1 and -6 real-time serotyping RT-PCR assays were evaluated using characterized biological samples collected during epidemics, epidemiological surveys [13,14,22] or naïve animals sampled in an EHDV-free area. The results, ranging from 98 to 100% for specificity and 99 to 100% for sensitivity, demonstrate that these tools were efficient in detecting EHDV and determining its serotype from a field blood sample. Moreover, the simultaneous amplification of an endogenous control gene (Beta-actin from wild or domestic ruminants) as an internal control attests to the quality of the analysed sample and the absence of PCR inhibitors [23]. If no Ct was obtained for the endogenous control gene when testing a ruminant blood sample, negative results for the EHDV target were not taken into account. In 2007, we already reported the use of this endogenous reporter gene [23]. However, beta-actin was not amplified in the same tube as the pathogen target (BTV). Duplexing is a real improvement for real-time RT-PCR assays. It optimises experiment efficiency by decreasing the volume of pipetted sample required and the cost of reagents, and by increasing the number of samples tested in the same run. The primers/probe set for all of the pan-EHDV and serotyping assays described above were also determined for use with the same cycling parameters, and can therefore be used in the same run.

EHDV infection is widespread in the USA, where it causes severe epidemics in wild *Cervidae*, particularly white-tailed deer (*Odocoileus virginianus*) [29]. According to Allison [9], EHDV is one of the most important viral diseases of white-tailed deer worldwide. In the last decade, EHDV has also been reported in Maghreb, in the Middle East [11], Africa [15], the Caribbean basin [14] and Reunion Island [13,22,29] especially associated with disease in cattle. In most infected areas, Bluetongue and EHD virus co-circulate, and mixed infections are reported. These reports highlight the need for efficient diagnostic tools to quickly detect and identify the causal agent. The pan-EHDV assay developed in this work was able to detect all the strains and biological samples included in the study, representing all seven EHDV serotypes, without any cross reaction with Bluetongue strains. Moreover, the seven serotyping assays gave good results in terms of analytical/diagnostic specificity and sensitivity, making them reliable tools for serotype identification.

In conclusion, the development of such molecular assays for EHDV diagnosis is very useful for disease control. It is crucial to have a better knowledge of the worldwide distribution of this virus and to add viral sequences to the data bank.

## References

[1] Huismans H, Bremer CW, Barber TL. The nucleic acid and proteins of epizootic haemorrhagic disease virus. Onderstepoort J Vet. 1979;46(2):95–104. . 233147

[2] Mecham JO, Dean VC. Protein coding assignment for the genome of epizootic haemorrhagic disease virus. J Gen Virol. 1988;69 (Pt 6):1255–62. Epub 1988/06/01. . 3133450 10.1099/0022-1317-69-6-1255

[3] Mertens PP, Brown F, Sangar DV. Assignment of the genome segments of bluetongue virus type 1 to the proteins which they encode. Virology. 1984;135(1):207–17. Epub 1984/05/01. . 6328750 10.1016/0042-6822(84)90131-4

[4] Belhouchet M, Mohd Jaafar F, Firth AE, Grimes JM, Mertens PP, Attoui H. Detection of a fourth orbivirus non-structural protein. PLoS One. 2011;6(10):e25697 10.1371/journal.pone.0025697 22022432 PMC3192121

[5] Ratinier M, Caporale M, Golder M, Franzoni G, Allan K, Nunes SF, et al Identification and characterization of a novel non-structural protein of bluetongue virus. PLoS Pathog. 2011;7(12):e1002477 10.1371/journal.ppat.1002477 22241985 PMC3248566

[6] Anthony SJ, Maan S, Maan N, Kgosana L, Bachanek-Bankowska K, Batten C, et al Genetic and phylogenetic analysis of the outer-coat proteins VP2 and VP5 of epizootic haemorrhagic disease virus (EHDV): Comparison of genetic and serological data to characterise the EHDV serogroup. Virus Res. 2009;145(2):200–10. 10.1016/j.virusres.2009.07.012 19632281

[7] Chalmers GA, Vance HN, Mitchell GJ. An outbreak of epizootic hemorrhagic disease in wild ungulates in alberta. Wildlife Diseases. 1964;6.

[8] Shope RE, MacNamara LG, Mangold R. Epizzotic Haemorragic Disease ofdeer. New Jersey Outdoors. 1955;November 16:21.

[9] Allison AB, Goekjian VH, Potgieter AC, Wilson WC, Johnson DJ, Mertens PPC, et al Detection of a novel reassortant epizootic hemorrhagic disease virus (EHDV) in the USA containing RNA segments derived from both exotic (EHDV-6) and endemic (EHDV-2) serotypes. J Gen Virol. 2010;91(2):430–9. 19828758 10.1099/vir.0.015651-0

[10] Sugiyama M, Hirayama N, Sasaki H, Sugimura T, Minamoto N, Kinjo T. Antigenic relationship among strains of Ibaraki virus and epizootic haemorrhagic disease virus studied with monoclonal antibodies. Res Vet Sci. 1989;46(2):283–5. Epub 1989/03/01. . 2539639

[11] Temizel EM, Yesilbag K, Batten C, Senturk S, Maan NS, Clement-Mertens PP, et al Epizootic hemorrhagic disease in cattle, Western Turkey. Emerg Infect Dis. 2009;15(2):317–9. Epub 2009/02/06. 19193283 10.3201/eid1502.080572 PMC2662652

[12] Yadin H, Brenner J, Bumbrov V, Oved Z, Stram Y, Klement E, et al Epizootic haemorrhagic disease virus type 7 infection in cattle in Israel. Vet Rec. 2008;162(2):53–6. Epub 2008/01/15. . 18192658 10.1136/vr.162.2.53

[13] Sailleau C, Zanella G, Breard E, Viarouge C, Desprat A, Vitour D, et al Co-circulation of bluetongue and epizootic haemorrhagic disease viruses in cattle in Reunion Island. Vet Microbiol. 2012;155(2–4):191–7. 10.1016/j.vetmic.2011.09.006 22005178

[14] Viarouge C, Lancelot R, Rives G, Breard E, Miller M, Baudrimont X, et al Identification of bluetongue virus and epizootic hemorrhagic disease virus serotypes in French Guiana in 2011 and 2012. Vet Microbiol. 2014;174(1–2):78–85. Epub 2014/10/11. 10.1016/j.vetmic.2014.09.006 . 25301282

[15] Toye PG, Batten CA, Kiara H, Henstock MR, Edwards L, Thumbi S, et al Bluetongue and epizootic haemorrhagic disease virus in local breeds of cattle in Kenya. Res Vet Sci. 2013;94(3):769–73. Epub 2012/12/25. 10.1016/j.rvsc.2012.11.001 23261160 PMC3632752

[16] Maan NS, Maan S, Nomikou K, Johnson DJ, El Harrak M, Madani H, et al RT-PCR assays for seven serotypes of epizootic haemorrhagic disease virus & their use to type strains from the Mediterranean region and North America. PloS one. 2010;5(9). Epub 2010/09/24. 10.1371/journal.pone.0012782 20862243 PMC2941451

[17] Breard E, Belbis G, Viarouge C, Riou M, Desprat A, Moreau J, et al Epizootic hemorrhagic disease virus serotype 6 experimentation on adult cattle. Res Vet Sci. 2013;95(2):794–8. Epub 2013/08/01. 10.1016/j.rvsc.2013.06.026 . 23899717

[18] Clavijo A, Sun F, Lester T, Jasperson DC, Wilson WC. An improved real-time polymerase chain reaction for the simultaneous detection of all serotypes of Epizootic hemorrhagic disease virus. J Vet Diagn Invest. 2010;22(4):588–93. 20622230 10.1177/104063871002200414

[19] Wilson WC, Hindson BJ, O'Hearn ES, Hall S, Tellgren-Roth C, Torres C, et al A multiplex real-time reverse transcription polymerase chain reaction assay for detection and differentiation of Bluetongue virus and Epizootic hemorrhagic disease virus serogroups. J Vet Diagn Invest. 2009;21(6):760–70. 19901276 10.1177/104063870902100602

[20] Sun F, Cochran M, Beckham T, Clavijo A. Molecular typing of epizootic hemorrhagic disease virus serotypes by one-step multiplex RT-PCR. J Wildl Dis. 2014;50(3):639–44. Epub 2014/05/09. 10.7589/2013-11-302 . 24807175

[21] OIE. Manual of Diagnostic Tests and Vaccines for Terrestrial Animals 2014.

[22] Cetre-Sossah C, Roger M, Sailleau C, Rieau L, Zientara S, Breard E, et al Epizootic haemorrhagic disease virus in Reunion Island: evidence for the circulation of a new serotype and associated risk factors. Vet Microbiol. 2014;170(3–4):383–90. Epub 2014/03/19. 10.1016/j.vetmic.2014.02.007 . 24636165

[23] Toussaint JF, Sailleau C, Breard E, Zientara S, De Clercq K. Bluetongue virus detection by two real-time RT-qPCRs targeting two different genomic segments. J Virol Methods. 2007;140(1–2):115–23. Epub 2007/01/02. 10.1016/j.jviromet.2006.11.007 . 17196266

[24] Ballagi-Pordany A, Belak S. The use of mimics as internal standards to avoid false negatives in diagnostic PCR. Mol Cell Probes. 1996;10(3):159–64. Epub 1996/06/01. 10.1006/mcpr.1996.0022 . 8799368

[25] Clopper C, Pearson E. The use of confidence or fiducial limits illustrated in the case of the binomial. Biometrika. 1934;26:404–13.

[26] Schroeder ME, Johnson DJ, Ostlund EN, Meier J, Bounpheng MA, Clavijo A. Development and performance evaluation of a streamlined method for nucleic acid purification, denaturation, and multiplex detection of Bluetongue virus and Epizootic hemorrhagic disease virus. J Vet Diagn Invest. 2013;25(6):709–19. Epub 2013/10/05. 10.1177/1040638713503654 . 24091683

[27] Mertens P, Maan S, Samuel A, Attoui H. Orbivirus Reoviridae In: Fauquet CM, Mayo MA, Maniloff J, Desselberger U, Ball LA, editors London 2005;Virus Taxonomy VIIIth Report of the ICTV Elsevier/Academic Press:466–83.

[28] Savini G, Afonso A, Mellor P, Aradaib I, Yadin H, Sanaa M, et al Epizootic heamorragic disease. Res Vet Sci. 2011;91(1):1–17. 10.1016/j.rvsc.2011.05.004 21665237

[29] Breard E, Sailleau C, Hamblin C, Graham SD, Gourreau JM, Zientara S. Outbreak of epizootic haemorrhagic disease on the island of Reunion. Vet Rec. 2004;155(14):422–3. Epub 2004/10/29. . 15508843 10.1136/vr.155.14.422

